# Global and Site-Specific Changes in 5-Methylcytosine and 5-Hydroxymethylcytosine after Extended Post-mortem Interval

**DOI:** 10.3389/fgene.2016.00120

**Published:** 2016-06-23

**Authors:** Jeffrey A. Gross, Corina Nagy, Li Lin, Éric Bonneil, Marissa Maheu, Pierre Thibault, Naguib Mechawar, Peng Jin, Gustavo Turecki

**Affiliations:** ^1^Department of Psychiatry, McGill Group for Suicide Studies, Douglas Mental Health University Institute, McGill University, MontrealQC, Canada; ^2^Department of Human Genetics, Emory University School of Medicine, AtlantaGA, USA; ^3^Institute for Research in Immunology and Cancer, Université de Montréal, MontrealQC, Canada

**Keywords:** epigenetics, post-mortem interval, 5-methylcytosine, 5-hydroxymethylcytosine, stability

## Abstract

There has been a growing interest in the study of epigenetic mechanisms to elucidate the molecular bases of human brain-related diseases and disorders. Frequently, researchers utilize post-mortem tissue with the assumption that post-mortem tissue decay has little or no effect on epigenetic marks. Although previous studies show no effect of post-mortem interval on certain epigenetic marks, no such research has been performed on cytosine modifications. In this study, we use DNA from the brains of adult Sprague Dawley rats subjected to post-mortem intervals at room temperature, ranging from 0 to 96 h, to assess the stability of cytosine modifications, namely 5-methycytosine and 5-hydroxymethylcytosine. Our results indicate that neither global nor site-specific levels of 5-methycytosine and 5-hydroxymethylcytosine are affected by the post-mortem intervals we studied. As such, the use of post-mortem tissue to study cytosine modifications in the context of neurological or neuropsychiatric disorders is appropriate.

## Introduction

There has been growing interest in the investigation of epigenetic changes associated with disease states. Among pathological conditions of the brain, post-translational histone modifications, non-coding RNAs, and cytosine modifications have all been associated with developmental disorders, neurological diseases, psychiatric illnesses, and cancers, among others.

In particular, 5-methylcytosine (5mC) has been the most widely studied epigenetic modification. Research has shown 5mC to be central to the establishment of tissue-specific gene expression, cell differentiation, genomic imprinting, and X-inactivation. Recently, however, interesting research provided evidence showing that 5mC can be oxidized to 5-hydroxymethylcytosine (5hmC) in a reaction catalyzed by the ten–eleven translocation (TET) enzymes (Kriaucionis and Heintz, 2009; Tahiliani et al., 2009; Ito et al., 2010). This finding led to the discovery of additional oxidative products, namely 5-formylcytosine (5fC) and 5-carboxylcytosine (5caC). Together, these oxidative products of 5mC have led to the proposal of active DNA demethylation, where methylation is removed from a cytosine base by successive oxidations and base excision repair. Although clues are rapidly emerging, questions still remain on the dynamics of the cytosine demethylation pathway and the functions of these intermediate molecules. Nevertheless, growing evidence points to the involvement of 5mC and its oxidative products in human diseases and disorders.

To determine the function of cytosine modifications in human brain diseases, such as those related to neuropsychiatric, neurodevelopmental, and neurodegenerative phenotypes, many researchers must rely on post-mortem brain tissue to conduct their studies. Typically, post-mortem studies report post-mortem interval (PMI), which represents the amount of time between a subject’s death and collection and processing of the brain. It has previously been shown that DNA is stable across extended PMIs (Hynd et al., 2003), while the same is true for microRNAs (miRNAs) and some histone modifications (Huang et al., 2006; Stan et al., 2006; Nagy et al., 2015). It has also been hypothesized that the skull protects the brain from oxidative damage during extended PMIs (Hynd et al., 2003), which is of interest given that 5hmC, 5fC, and 5caC are oxidative products of 5mC. Although initial reports confirm that lengthy PMIs may not alter epigenetic landscapes, their effects on DNA demethylation products have yet to be determined. In this report, we provide evidence that these cytosine modifications are stable during post-mortem intervals and can reflect the actual status of 5mC and 5hmC at the time of death in post-mortem tissue.

## Materials and Methods

### Experimental Design

**FIGURE 1:**
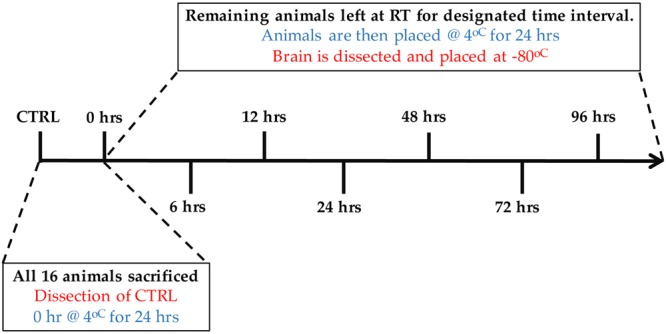
**Experimental design showing post-mortem intervals (PMIs) from 0 to 96 h.** All animals (*n* = 16, 2 per condition) were sacrificed at the same time. Brains from the control animals (CTRL) were dissected immediately following sacrifice, flash frozen and stored at -80°C. After being sacrificed, animals from the 0 h time point were placed immediately at 4°C for 24 h, followed by brain dissection and storage at -80°C. The remaining animals were left at room temperature for the designated PMIs (6–96 h), followed by 24 h at 4°C. Brains were dissected and stored at -80°C thereafter.

Sprague Dawley rats (Charles River Laboratories International, Wilmington, MA, USA) were selected as a model to test the effects of PMI. Young adult rats (60-days old, *n* = 16) were caged independently and allowed to habituate to the environment for one week. The animals were then sacrificed by gas asphyxiation and left in open air for various PMIs. In order to mimic natural biological circumstances, the rats were subjected, at room temperature, to PMIs ranging from 0 to 96 h, and a fixed time of 24 h at 4°C to simulate the time a human donor typically spends in the morgue before tissue collection. Eight different PMI time points (*n* = 2 per PMI) were investigated: Control (immediate dissection and flash freezing in isopentane cooled to -50°C), 0, 6, 12, 24, 48, 72, and 96h (**Figure 1**). All but the control animals, which were processed immediately after death with no PMI, were kept at 4°C for 24h before processing. After processing, all samples were stored at -80°C. This study was approved by the Douglas Hospital Research Centre’s Animal Research Committee (#04/15).

### DNA Extraction for LC–MS, ELISA, and 5hmC Capture

DNA was extracted from rat brain using a phenol:chloroform:isoamyl extraction protocol. Briefly, 40 mg of frozen tissue was homogenized in lysis buffer and incubated overnight with proteinase K. DNA was treated with RNAse A prior to phenol:chloroform:isoamyl extraction. NanoDrop 2000 spectrophotometer and Quant-IT PicoGreen (Thermo Scientific, Waltham, MA, USA, Cat. #: P7589) were used to assess the DNA purity and concentration.

### Liquid Chromatography–Mass Spectrometry (LC–MS)

One microgram of genomic DNA per sample was incubated with 5 units of the DNA Degradase Plus (Zymo Research, Irvine, CA, USA, Cat.# E2021) enzyme overnight at 37°C. Samples were loaded and separated on a homemade reversed-phase column (150 μm i.d. × 150 mm), with a gradient going from 0 to 10% B in 18 min and a 600 nl/min flow rate on an Easy nLC (Thermo Scientific, Waltham, MA, USA) connected to a Fusion Orbitrap (Thermo Scientific, Waltham, MA, USA). MS analysis consists of a PRM acquisition method for C, mC, and hmC, consisting, respectively, of a SIM scan at *m/z* at 228.1, 242.1, and 256.1, followed by tandem mass spectra for each transition at a normalized collision energy (NCE) of 30%. Ion chromatograms were extracted for fragment ions *m/z* 112.0505, 126.0661, and 142.0611.

### ELISA-Based Global 5mC and 5hmC Quantification

One hundred nanograms of DNA per sample were denatured, as per manufacturer’s guidelines, and added to Zymo Research ELISA kits (Zymo Research, Irvine, CA, USA, Cat. # D5325 and D5425 for 5mC, and 5hmC, respectively). Primary and secondary antibodies were added following manufacturer’s guidelines. Plates were allowed to develop at room temperature and absorbance was read at 405nm for both kits.

### DNA Extraction for EpiTYPER

DNA was extracted from 40 mg of frozen rat brain tissue using the Qiagen DNeasy Blood & Tissue kit (Qiagen Inc., Toronto, ON, Canada, Cat.# 69506). NanoDrop 2000 spectrophotometer (Thermo Scientific, Waltham, MA, USA) was used to assess the purity and concentration of the DNA. DNA was bisulfite converted using EpiTect Bisulfite Kit (Qiagen Inc., Toronto, ON, Canada, Cat.# 59104). Briefly, DNA was treated with sodium bisulfite, cycling between 95 and 60°C for 5 h. These conditions result in deamination of the cytosine into a uracil, except in the presence of a modified cytosine. The combination of 5mC and 5hmC was assessed using the EpiTYPER service platform from the McGill University Genome Quebec Innovation Centre. The Centre designed the primers for *h19* and *Igf2*, which are maternally and paternally imprinted regions, respectively, using EpiDesigner. Regional coordinates for *h19* and *Igf2* were chr1: 222,641,272–222,641,531 and chr1: 202,911,352–202,911,723, respectively.

### 5hmC Capture

5hmC from rat brain DNA was captured using selective chemical labeling, as previously described (Song et al., 2011). Final concentrations of captured DNA were measured using Qubit Fluorometric Quantitation (Life Technologies, Carlsbad, CA, USA). Site-specific levels of 5hmC were assessed by quantitative real-time polymerase chain reaction (qRT-PCR) on Applied Biosystems’ *7900HT* Fast Real-Time PCR System (Applied Biosystems, Carlsbad, CA, USA). SYBR green primers were tiled across the same h19 and Igf2 loci as the EpiTYPER analyses. Primer sequences are listed in **Table 1**. The 9600 emulation thermal cycle protocol was: 50°C for 2 min, 95°C for 10 min, and 40 repetitions of 95°C for 15 s and 60°C for 1 min. The data were extracted by relative quantification using Applied Biosystems’ SDS 2.4 and RQ Manager 1.2.1 software. Data were normalized using input DNA, which represents non-captured sheared DNA.

**Table 1:** Primer sequences for qRT-PCR following 5hmC capture.

Target	Forward primer	Reverse primer	Length (bp)
Ifg2–1	TTGTGGGCTCCATTGAGTTT	ACGGCGTCCGTCTCATTAT	118
Ifg2–2	AAACCCTCCTGTTTTTGCAG	ACACCCCTATCCCCAAGAGT	135
Ifg2–3	ACTCTTGGGGATAGGGGTGT	AAGCACCAACATCGACTTCC	104
Ifg2–4	GGAAGTCGATGTTGGTGCTT	CAAACTGAAGCGTGTCAACAA	114
h19–1	GGGCAGTGAAGGTGTAGCTG	GCCTCGCTCTCTAAACCTTTT	124
h19–2	TCGCTGCACTGACCTTCTAA	CCGAGACGATGTCTCCTTTG	119
h19–3	GAAAGGCAGGACAGTTAGCAA	CAGCCCTGCACCTCTTCTAT	129

### Statistical Analyses

All statistical tests were performed using GraphPad Prism 6. The specifics of each test are outlined in the results section. Statistical significance was set at *p* < 0.05. Error bars represent Mean ± S.E.M.

## Results

DNA cytosine methylation is considered a highly stable nucleotide modification that remains unchanged over time (Barrachina and Ferrer, 2009), largely due to the strong covalent bond between the methyl group and the 5′ carbon of cytosine. Indeed DNA methylation was previously shown to withstand various changes in pH with no differences observed even in extremes (Ernst et al., 2008). Since this covalent bond is maintained during cytosine oxidation, the stability of the demethylation intermediates should also remain stable across increasing PMI. To evaluate this hypothesis, the primary cytosine modifications, 5mC and 5hmC, were tested both globally and at specific loci for 8 different PMIs.

### Global Levels of 5mC and 5hmC

**FIGURE 2:**
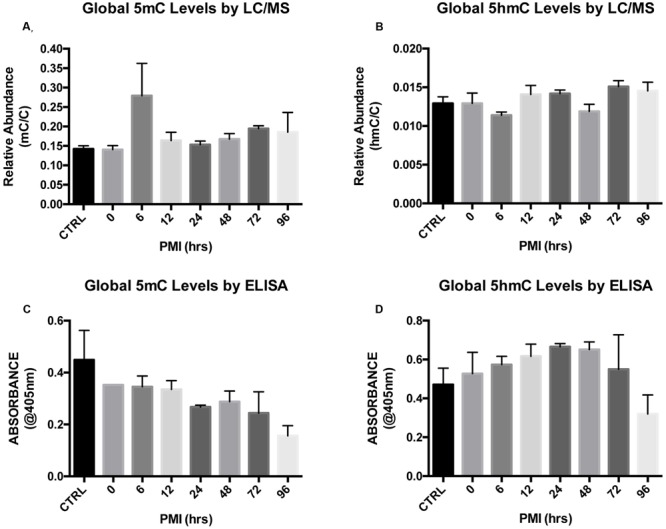
**Global levels of 5mC and 5hmC show no differences across increasing PMI. (A,B)** Global levels of 5mC **(A)** and 5hmC **(B)** were measured by LC/MS (*H* = 10.36; *p* = 0.1690 and *H* = 11.88; *p* = 0.1044, respectively). **(C,D)** LC/MS results for 5mC **(C)** and 5hmC **(D)** were validated using ELISA-based quantification (*H* = 10.28; *p* = 0.1733 and *H* = 7.103; *p* = 0.4182, respectively). Dunn’s multiple comparisons tested individual PMIs against the CTRL. *n* = 2 per PMI. Significance set at *p* < 0.05.

We first explored global levels of each cytosine modification by LC–MS. Two subjects per time point were run in duplicate. Kruskal–Wallis ANOVA followed by Dunn’s multiple comparison tests showed no effect of PMI on global 5mC (*H* = 10.36; *p* = 0.1690) or 5hmC (*H* = 11.88; *p* = 0.1044) levels (**Figures 2A,B**). As an alternative method to detect global levels, we used absorbance-based ELISA kits with antibodies specific to 5mC and 5hmC. Kruskal–Wallis ANOVA followed by Dunn’s multiple comparison tests confirmed that increasing PMI does not alter global 5mC (*H* = 10.28; *p* = 0.1733) or 5hmC (*H* = 7.103; *p* = 0.4182) levels (**Figures 2C,D**).

### Site-Specific Levels of 5mC and 5hmC

**FIGURE 3:**
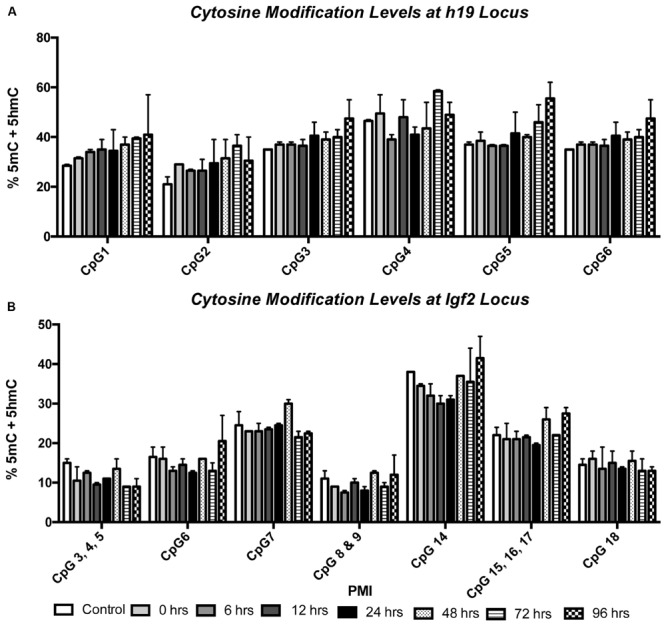
**Site-specific levels of cytosine modifications across the h19 and Igf2 loci remain stable with increasing PMI.** EpiTYPER was used to analyze the percent of methylation + hydroxymethylation at the h19 **(A)** and Igf2 **(B)** loci (*F*_(35,42)_ = 0.3603; *p* = 0.9987 and *F*_(42,49)_ = 0.6150; *p* = 0.9453, respectively). Dunnett’s multiple comparisons tested %5mC + 5hmC of individual PMIs against the CTRL for each CpG. *n* = 2 per PMI. Significance set at *p* < 0.05.

**FIGURE 4:**
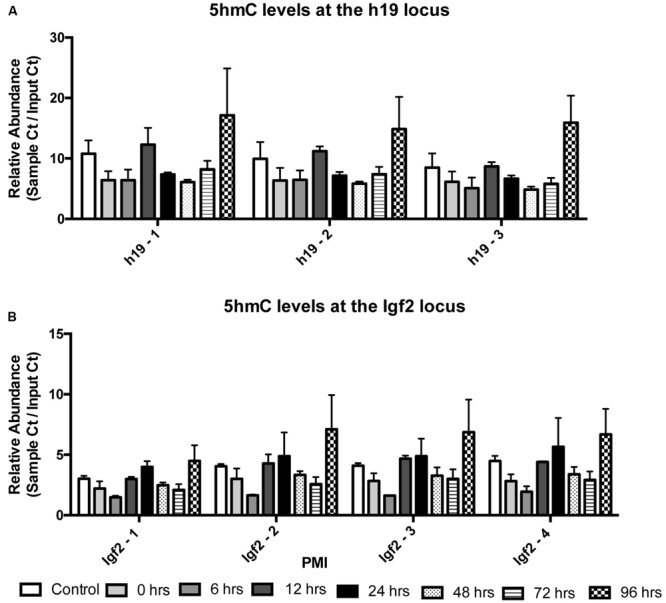
**Site-specific levels of 5hmC across the h19 and Igf2 loci remain stable with increasing PMI.** 5hmC capture by selective chemical labeling was used to analyze relative 5hmC levels (sample *C*_t_/Input *C*_t_) at the h19 **(A)** and Igf2 **(B)** loci (*F*_(14,24)_= 0.07283; *p* > 0.9999 and *F*_(21,32)_ = 0.1145; *p* > 0.9999, respectively). Dunnett’s multiple comparisons tested relative 5hmC levels of individual PMIs against the CTRL for each amplicon within the region of interest. *n* = 2 per PMI. Significance set at *p* < 0.05.

We also wanted to know whether 5mC and 5hmC show loci-specific stability. To do so, we investigated two genes, *h19* and *Igf2*, both of which are known to undergo parental imprinting in humans and rats. Bisulfite conversion followed by EpiTYPER was used to assess the combination of 5mC and 5hmC, while levels of 5hmC were assessed using 5hmC capture followed by qRT-PCR. Two-way ANOVA showed no significant interaction between CpG cytosine modification and PMI for the h19 (*F*_(35,42)_ = 0.3603; *p* = 0.9987) (**Figure 3A**) or Igf2 (*F*_(42,49)_ = 0.6150; *p* = 0.9453) (**Figure 3B**) loci, while Dunnett’s multiple comparison test showed no difference in CpG cytosine modification between the control and increasing PMIs. Similarly, two-way ANOVA also showed no significant interaction between locus-specific 5hmC and PMI for h19 (*F*_(14,24)_ = 0.07283; *p* > 0.9999) (**Figure 4A**) or Igf2 (*F*_(21,32)_ = 0.1145; *p* > 0.9999) (**Figure 4B**), while Dunnett’s multiple comparison test showed no difference in 5hmC levels between the control and increasing PMIs.

## Discussion

The use of post-mortem tissue is an invaluable resource to investigate biological processes in the brain. However, using tissue collected postmortem may present certain limitations. Of great interest is how the PMI may affect the quality of tissue and our ability to generate reliable data. As cytosine modifications are a focus of much of the research in the field of epigenetics, it is of paramount importance to understand whether PMI alters the DNA demethylation pathway. In this study, we show that global and loci-specific levels of cytosine modifications remain largely unchanged across increasing PMIs. This data lends weight to the notion that cytosine modifications are stable and can be accurately assessed in postmortem research.

Our findings are reassuring considering the longevity of DNA integrity after death. Interestingly, it was recently determined that the half-life of DNA is 521 years (Allentoft et al., 2012). Although this study referred to a specific mitochondrial DNA sequence from fossils, research suggests a distinct stability of DNA, especially from the brain (Bar et al., 1988). These findings are pertinent when considering the potential for oxidative damage occurring during death. Since oxidation of 5mC results in downstream modifications to cytosine, it may be presumed that these modifications are susceptible to degradation following death. Our results counter this presumption and show an impressive stability of the tested cytosine modifications. Our findings are more in line with the proposal that cortical DNA is particularly stable, as it is protected by the skull from environmental contamination (Bar et al., 1988; Hynd et al., 2003).

This study is not without several methodological limitations. To best mimic human conditions, Sprague Dawley rats were selected as a model for PMI as their genetic backgrounds are similar to humans. Although it would have been ideal to also take into account the effects of agonal conditions, such as long hospital stays, comas, or hypoxic states, subjecting the rats to these antemortem conditions would not be ethical. In addition, when studying brain tissue exposed to long post-mortem intervals, distinguishing brain anatomy is quite challenging. In this study, we used similar sections from the frontal region of each brain, although the exact region cannot be reported. Furthermore, although bisulfite conversion does not distinguish between 5mC and 5hmC (Huang et al., 2010) for site-specific methylation analysis, we saw no differences in 5hmC through the accompanying 5hmC capture. As such, 5hmC is unlikely to represent a significant variation in the EpiTYPER analyses, thereby making them generalizable to 5mC as well. Another potential limitation was our inability to quantify 5fC or 5caC, however, given the very low abundance of these demethylation intermediates across tissue types (Ito et al., 2011), this should not come as a surprise. In fact, previous studies utilized thymine DNA glycosylase (TDG) knockdowns or knockouts to increase the levels of 5fC and 5caC (Shen et al., 2013; Song et al., 2013; Wu et al., 2014; Neri et al., 2015). Nevertheless, given the inherent stability of DNA, along with the stability of 5mC and 5hmC, we hypothesize that 5fC and 5caC would not be affected by increasing PMI. To confirm this hypothesis, further studies could first create a TDG knockout animal, then continue with the PMI protocol described in this manuscript.

Taken together, the results presented here may enhance confidence in future research studying cytosine modifications in post-mortem samples. Undoubtedly, the continuing debate regarding the function of these modifications as DNA demethylation intermediates (He et al., 2011; Shen et al., 2013) or as a transcriptional regulators (Mellen et al., 2012; Lister et al., 2013; Wen et al., 2014; Gross et al., 2015) will require more in-depth analyses using post-mortem tissue. Furthermore, the stability of these modifications across PMIs allows researchers to continue elucidating the function of cytosine modifications in brain-related diseases and disorders.

## Author Contributions

JG, CN, and GT designed the study; JG and CN performed all the analyses; CN, MM, and NM performed the animal work; JG, EB, and PT performed the LC–MS; JG and CN performed the ELISAs; JG and CN performed EpiTYPER; JG, LL, and PJ performed 5hmC capture; All authors wrote and approved the manuscript.

## Conflict of Interest Statement

The authors declare that the research was conducted in the absence of any commercial or financial relationships that could be construed as a potential conflict of interest.
